# Emerging Strategies Mold Plasticity of Vegetable Plants in Response to High Temperature Stress

**DOI:** 10.3390/plants11070959

**Published:** 2022-04-01

**Authors:** Wen-Feng Nie, Enjie Xing, Jinyu Wang, Yueying Mao, Xiaotao Ding, Jianfei Guo

**Affiliations:** 1 Department of Horticulture, College of Horticulture and Plant Protection, Yangzhou University, Yangzhou 225009, China; xej2021@163.com (E.X.); wjy160123@163.com (J.W.); paomianwan@163.com (Y.M.); 2 Shanghai Key Laboratory of Protected Horticultural Technology, Horticulture Research Institute, Shanghai Academy of Agricultural Sciences, Shanghai 201403, China; 3 Shenzhen Branch, Guangdong Laboratory of Lingnan Modern Agriculture, Genome Analysis Laboratory of the Ministry of Agriculture and Rural Affairs, Agricultural Genomics Institute at Shenzhen, Chinese Academy of Agricultural Sciences, Shenzhen 518120, China

**Keywords:** global warming, vegetables, gene editing, breeding, epigenetic modification, plant plasticity

## Abstract

As a result of energy consumption and human activities, a large amount of carbon dioxide emissions has led to global warming, which seriously affects the growth and development of plants. Vegetables are an indispensable part of people’s diet. In the plant kingdom, a variety of vegetables are highly sensitive to climate change. For them, an increase of just a few degrees above their optimum temperature threshold can result in a loss of yield and quality. Emerging strategies such as practice management and breeding varieties in response to above-optimal temperatures are critical for abiotic stress resistance of vegetable crops. In this study, the function and application of multiple strategies, including breeding improvement, epigenetic modification directed generation of alleles, gene editing techniques, and accumulation of mutations in multigenerational adaptation to abiotic stress, were discussed in vegetable crops. It is believed to be meaningful for plants to build plasticity under high temperature stress, thus generating more genetic structures for heat resistant traits in vegetable products.

## 1. Introduction

Climate change is a natural process caused by both internal and external factors, in which the continuous change of atmospheric composition largely affects the efficiency of land use. Moreover, climate change and its interaction with intensive agricultural management may lead to nitrogen losses, which in turn constrains environmental and human health at local, regional and global scales [1]. Global warming is a typical example of climate change, which has a profound impact on agricultural production. Scientists suggest that limiting global warming to 1.5 °C could reduce the risk of 2 °C warming by half for plants and animals, and by about 66% for insects [2,3], indicating that plants are highly sensitive to changes in ambient temperature. Hence, climate change poses challenges to practice management. In practice management, understanding how crops respond to climate change is critical to prevent damage from temperature change. Plants responding to high temperature stress depend on the degree of overtemperature, duration, plant genotypes, and other co-existing environmental conditions. Heat generally impairs photosynthetic activity, germination, and reproduction and yield [4], while the complicated transcriptional regulatory network, post-translational regulation of the transcription factors, epigenetic mechanisms, and non-coding RNAs are involved in high temperature induced responses and stress memory [5].

High temperature stress is a main limiting factor of the yield of vegetables grown in greenhouses in summer [6]. In fact, the sensitivity of plants to temperature increases is much higher than laboratory data shows [7] and may even be around 1 °C [8], greatly increasing the complexity and difficulty of planning and adjusting strategies to prevent crop yield loss. Vegetable products are an important part of the human diet, and many of them are temperature-dependent. For example, high temperatures can lead to early flowering of non-heading Chinese cabbage (*Brassica rapa ssp. chinensis*) [9] and a significant reduction in flower number and seed production per plant [10]. Moreover, it affects the leaf pigmentation and quality of *Brassica oleracea* L. [11], the yield of potato [12], as well as fruit setting rate in *Solanum lycopersicum* [13]. 

Vegetables are essential to global food supply and sensitive to heat stress. Higher than optimal growing temperatures, no matter day or night, can significantly affect crop yields, making heat stress a major challenge for horticultural crop production. For these reasons, how to cope with high temperature stress in vegetable production has been a hot topic of scientific research in recent years. 

## 2. Emerging Strategies in Vegetable Practice Management

### 2.1. Genetic Breeding Resource

In breeding, abundant diversity of genetic resources is of great significance for biological evolution to cope with abiotic stress [14,15,16,17]. Cappetta et al. evaluated the phenotypic characteristics of quantitative and qualitative traits by applying heat stress treatment to self-crossing F4 segregated populations of heat tolerant tomato varieties, and predicted several potential loci that may be involved in high temperature response by calculating the effect of a single nucleotide polymorphism (SNP)-dependent variation and combining with quantitative trait locus (QTL) analysis. This, to some extent, reveals a genome-selective (GS)-dependent approach that can control interactions between plants and high temperatures [13]. Lu and colleagues then found that after exposure to extreme heat and moderate warming, mutation rates of single-nucleotide variations (SNVs) and small indoles were increased in *Arabidopsis thaliana* multigenerational accumulation plants, which is associated with changes in epigenetic modifications, such as DNA methylation levels [18]. These studies and advances provide insights and guidance on genetic and epigenetic structures as well as correlations between different biological traits (i.e., yield and growth-dependent biomass) under high temperature stress. The optimized genetic prediction model is plausible as a valuable strategy to accelerate the development of heat tolerance in tomato fruits with high yield and soluble solids content [13], which is superposed with the potential impact of epigenetic improvement of breeding resources to further enrich vegetable food traits through the crop practice management [19,20]. 

In recent decades, in order to overcome the defects caused by extreme temperature on yield and quality of vegetable products, a large number of studies have been carried out, such as the application of plant hormones [21,22,23] and the use of grafting to change rootstocks [24]. In potatoes, gibberellin may be involved in thermal sprouting and dormancy release caused by heat shock in summer; thermal sprouting and postharvest sprouting share common target genes and similar gene expression patterns [25], showing the irresistibility of high temperature induced quality loss of potatoes. Although the quality and performance of vegetables in high temperatures summer environment have been partially improved, further attempts of genetic breeding should be made to cultivate more heat-resistant vegetables.

### 2.2. Gene Editing Technology

Gene regulatory networks are central to the understanding of all biological processes, including those that determine important crop traits such as yield, quality, and resistance to biological and abiotic stresses, which are sensitive to high temperature stimuli [8]. The application of gene editing techniques to promote germplasm improvement has been demonstrated in some crops [26,27,28]. Clustered regularly interspaced short palindromic repeats (CRISPR)/ CRISPR-associated protein (Cas) technology in genome editing includes prime editing [29], base editing [30], tissue-specific editing [31], epigenome editing [32], and inducible genome editing [33], which can be used as the strategies to obtain resistant varieties that can tolerate high-temperature stress [34]. For instance, by CRISPR/Cas9-based gene editing, *TaMBF1c* was confirmed to have a positive role in heat response in wheat [35], suggesting that the overexpression of homolog genes of *MBF1c* in vegetable crops could be considered as a method for the selection of resistant varieties. In plants, the expression of most heat shock proteins (HSPs) is transcriptionally regulated by heat shock transcription factors (HSFs) in response to higher temperatures, thus to minimize the damages caused by heat stress (HS) [36]. Hence, CRISPR/ CAS9 can facilitate the study on redundancy function of *HSPs* or *HSFs* genes by the simultaneous alteration of multiple genes [37]. The efficient establishment of tomato transformation system mediated by *Agrobacterium tumefaciens* also promoted the superiority of gene editing technology to be well reflected in tomato [38,39,40]. Through meta quantitative trait loci (MQTL) analysis and screening, several QTL associated with heat tolerance traits (e.g., pollen viability, number of pollens, number of flowers, style protrusions, style lengths) were identified [41], hinting that these QTLs could be targeted to perform genomic selection and breeding techniques including genome editing and molecular breeding to improve heat tolerance in tomato plants and fruits. As the system of transgenic technology has been gradually established in many other vegetable varieties [42,43], using CRISPR/cas9 technology to develop genetic resources (e. g. knocking out the corresponding genomic regions of non-coding RNAs targeting *HSFs*) and enhance the plasticity of vegetable varieties under high temperature stress has become a feasible method of molecular breeding in agricultural practice.

### 2.3. Interference by Epigenetic Modifications

Under the changeable growth environment, plant cells have developed complicated gene regulatory networks [5,44], including transcriptional level regulation involving multiple transcription factors [45] and post-transcriptional modification [46]. DNA mutations in the *Arabidopsis* genome were isolated after multiple generations of high temperature exposure and DNA methylation was found to play a role in the mutation process at high temperatures. Moreover, natural antisense transcripts (NATs) NAT398b/c inhibit microRNA398 biogenesis and reduce plant thermal tolerance via a regulatory loop mechanism [47]. These results suggest that evolution-based environmental changes may be altered by epigenetic modifications that affect the plant genome and epigenome. Post-transcriptional modification of genes can be achieved by affecting chromatin structure, histone modification, DNA methylation, histone variation and non-coding RNA, demonstrating the complexity of gene regulation mechanisms [48]. However, there are few reports on the specific mechanisms of histone variation and chromatin structure in response to mild hypothermia in plants, and similar regulatory mechanisms are still unclear in horticultural crops such as tomato. Epigenetic modification enriches the diversity of genetic information in vegetable crops [19]. The evolution of heat-induced SNPs accumulation is dynamically regulated by DNA methylation [18], suggesting that epigenetic modification and environmentally induced SNP-dependent genetic selection should be considered synergistically [13].

### 2.4. Possible Opening Avenues

In addition to breeding techniques and epigenetic modification dependent epialleles that have been used to improve tolerance to high temperature stress, several new approaches have been developed recently to promote biological evolution and adaptation to high temperature stress, such as beneficial interactions between microorganisms and plant hosts, and single spectral dependent light regulation. The aboveground structure of plants is very important for the yield and shelf life of horticultural products [19]. Different wavelengths of light have different effects on plants. For example, red light can transform biologically inactive photochrome Pr into biologically active photochrome Pfr, thus achieving maximum absorbance under far-infrared (FR) light, while blue light can activate the activities of cryptochrome and phototropin, thus accumulating the excited states of photosynthetic pigments [49].The phytochrome B (phyB) photoreceptors participate in temperature perception through its temperature-dependent conversion, specifically, the reversal from the active Pfr state to the inactive Pr state, and the bioactive Pfr form of phyB is converted to its inactive Pr form at high ambient temperatures [50,51,52]. On the other hand, blue light inhibits heat-mediated hypocotyl elongation through cryptochrome (CRY1) [53]. These studies suggest that photoreceptors are involved in high temperature induced thermomorphogenesis in plants, which is critical to the vegetative stage, flowering and reproductive development. With the revelation of the function of specific spectral and the application of light-emitting diodes (LEDs) in horticultural facilities [54], spectrum-dependent heat stress resistance should also be considered in practical management. 

**Figure 1:**
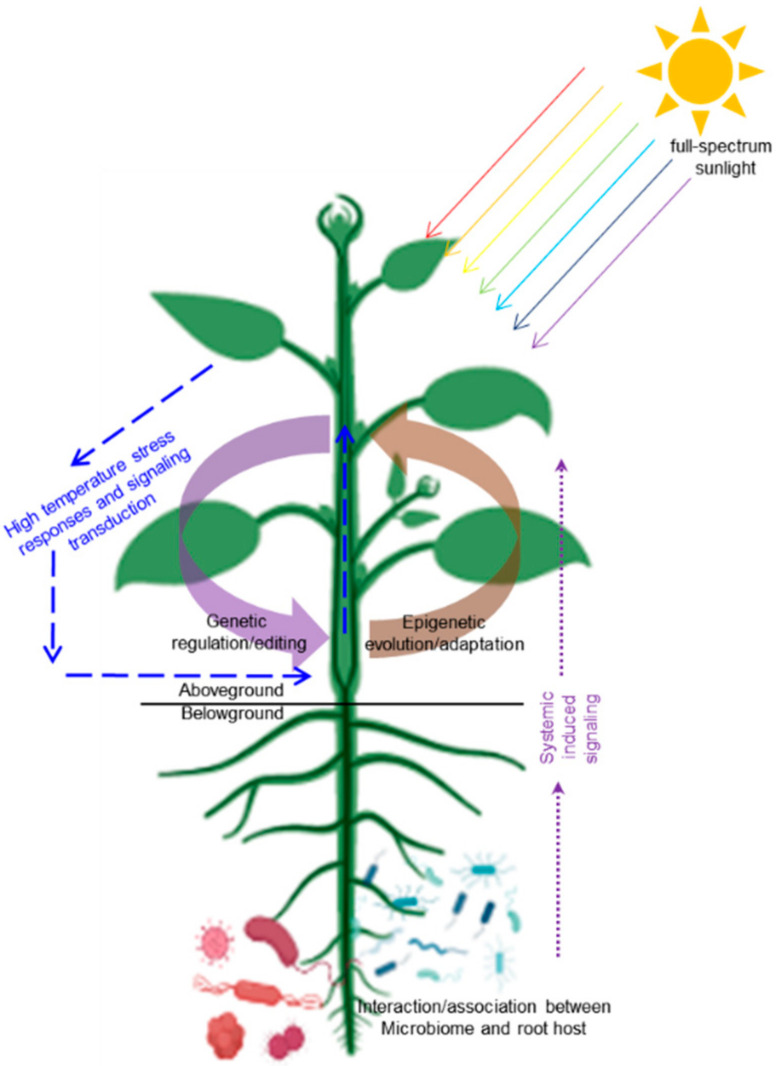
Schematic model showing that genetic and epigenetic dependent regulations/adaptations in molding the plasticity of vegetable plants responding to high temperature stress. The figure is created with bioRender.com with a few modifications.

Higher ambient temperatures can promote communication between viruses and infected hosts, thus affecting plant growth and agricultural productivity [55]. More generally, the high temperatures and humidity of the surrounding environment can induce bacterial disease in the roots of plants, which greatly reduces production. Cucumber fusarium wilt caused by *Fusarium oxysporum* occurred frequently in greenhouses with increasing temperature in summer. High temperature is conducive to virus transmission and systematic infection in cucumber plants [56], so it is necessary to prevent biohazards caused by high temperature. DNA methylation regulates the root microbiome, and exudates released by plant roots recruit beneficial microorganisms to promote growth and immunity in plants such as *Arabidopsis* and tomato [57]. This indicates that plants can improve their defense against pathogens via the interaction between microorganisms and roots [58,59], which can also constrain the damage to vegetable yield caused by rising ground temperature to a certain extent (Figure 1).

## 3. Discussion

Developing tomato varieties that produce higher yields at higher temperatures is a valuable strategy to combat global warming. To this end, a coordinated combination of multiple strategies, including breeding improvement [60,61], epigenetic modification directed generation of epialleles [62], gene editing techniques [30], and cumulative mutations of multiple generations adapted to abiotic stress [18], is likely to shape the plasticity of vegetable plants under heat stress, which can produce more heat resistant genetic structure in vegetable products in future.

A comprehensive understanding of the molecular mechanisms that determine the relationships between important agronomic traits will help further promote breeding techniques and research to shape tomato plasticity under high temperature stress [13]. The frequent occurrence of global temperature extremes has also greatly upset the balance of carbon dioxide metabolism. Temperature changes are caused by diurnal and seasonal variations as well as an increase in global average temperature due to climate change [8]. In addition, vegetables and their products are grown for long-term consumption. In that case, some vegetable crops are grown in seasons that are not suitable for them. Among the growth conditions, high temperature easily leads to the failure of fruit setting, early flowering and senescence of leaf-vegetables, as well as promoting the growth of branches and leaves [13]. However, high temperatures are reported to have a positive effect on flavor, as broccoli harvested during the hot summer months tastes good [60]. Even if this observation is broad and conservative in other vegetable crops, how to balance yield and quality still needs to be considered. In addition, a small increase in nighttime temperature destroys the tight temporal coordination between internal molecular events and the environment, thus reducing grain yield and quality [63], suggesting that an appropriate diurnal temperature difference is conducive to the normal growth and development of plants. This facilitates the accumulation of nutrients and the reduction of respiratory expenditure. Increased nighttime temperature leads to loss of product yield and quality, which is consistent with the concept that diurnal temperature difference determines the quality of many vegetables, such as the accumulation of carotenoids, sugars, antioxidants and ascorbic acid in vegetables and fruits [64]. This indicates that the negative effects of nighttime high temperatures also need to be taken seriously.

Although short-term regulation can be easily achieved in management practice through spectral dependent regulations and rhizosphere microbial interactions, genetic and epigenetic regulatory/adaptive capacities of vegetable species should be fully developed. This is because, previous studies have shown that when deciding on a global scale plant biomass and crop yield response to global warming, species and genotype-based intrinsic factors (evolutionary history) play a more important role than the external factors, including the experimental process and environmental conditions such as temperature, light, and their interaction with plant hormones [16,60]. As a response to current global warming, the maximum potential abundance of northern plant species is constantly changing [65], suggesting that interspecific differences directly contribute to the effects of global warming. Above all, a variety of strategies including breeding improvement methods, enriched breeding materials, epigenetic modification directed generation of epi-alleles, gene editing techniques and multigenerational adaption to abiotic stresses with accumulated mutations, can be applied for deciphering the response of consumer-demanded vegetable traits at different temperatures and facilitating the exploration of new cultivars and/or varieties adapted to climate change. As schematically shown in Figure 1, we present our understanding and considerations on how to improve the response of vegetable plants to high ambient warming by exploring the genetic and epigenetic regulatory/adaptive regulations of vegetable species.
